# Gametocytes: insights gained during a decade of molecular monitoring

**DOI:** 10.1016/j.pt.2008.08.001

**Published:** 2008-11

**Authors:** Hamza A. Babiker, Petra Schneider, Sarah E. Reece

**Affiliations:** 1 Biochemistry Department, Faculty of Medicine, Sultan Qaboos University, Alkhod, PO Box 35, Muscat, Code 123, Oman; 2 Institutes of Evolution, Immunology and Infection Research, School of Biological Sciences, University of Edinburgh, EH9 3JT, UK

## Abstract

In vertebrate hosts, malaria parasites produce specialized male and female sexual stages (gametocytes). Soon after being taken up by a mosquito, gametocytes rapidly produce gametes and, once mated, they infect their vector and can be transmitted to new hosts. Despite being the parasite stages that were first identified (over a century ago), gametocytes have remained elusive, and basic questions remain concerning their biology. However, the postgenomic era has substantiated information on the specialized molecular machinery of gametocytogenesis and expedited the development of molecular tools to detect and quantify gametocytes. The application of such highly sensitive and specific tools has opened up novel approaches and provided new insights into gametocyte biology. Here, we review the discoveries made during the past decade, highlight unanswered questions and suggest new directions.

## The dawn of molecular detection

Malaria is a debilitating disease that is responsible for between one and three million deaths annually, across tropical and subtropical climatic zones. The causative agents, *Plasmodium* parasites, replicate asexually in the blood of their vertebrate hosts, and a proportion of these asexually produced parasites differentiate into male and female sexual stages (gametocytes). Whereas the asexual stage in the life cycle of the parasite is responsible for clinical disease, gametocytes are responsible for transmission from host to vector. When taken up in the bloodmeal of a vector, male and female gametocytes immediately leave their red blood cells and produce gametes, which then mate and differentiate into stages that are infective to mosquitoes. Parasites then progress through several developmental stages in their vector, culminating in sporozoite stages that move to the salivary glands, ready to be transmitted to new hosts. Although sexual reproduction in *Plasmodium* parasites was discovered over a century ago, key questions about gametocyte biology remain. For example, the investment in gametocytes (the density of gametocytes relative to asexual forms, or conversion rate) and their sex ratio (the proportion of gametocytes that are male) both vary extensively across and within parasite species, populations and individual infections. Progress is being made in uncovering the genes and proximate mechanisms responsible [1–4], but the ultimate evolutionary explanations for this variation remain poorly understood [5–8].

Given the essential role that gametocytes have in transmission and the drive to develop clinical interventions that disrupt sexual reproduction, why are there still fundamental unanswered questions about their biology? This is, at least in part, because gametocytes occur at much lower densities than asexual parasites, which makes them more difficult to detect. However, with the development of a gametocyte-specific PCR assay for *P. falciparum* in 1999 [9], the elusive gametocytes became detectable with greater sensitivity. Over the past decade [10], the continued development of molecular techniques has provided assays that are sensitive enough to: (i) detect and quantify gametocytes at low densities [9,11–13]; (ii) differentiate gametocytes at early and late stages of development [14]; (iii) quantify gametocytes produced by different parasite genotypes in multi-genotype infections [15,16]; (iv) determine the expression pattern of sexual-stage-specific genes [17]; and (v) distinguish between male and female gametocytes, including those produced by different genotypes in multi-genotype infections [18]. These assays have stimulated major lines of research, from epidemiological and population-level surveys of gametocyte carriers and their transmission potential to the performance and behaviour of individual parasite genotypes during infections and the assessment of transmission outcomes after anti-malarial therapy. Here, we provide an overview of the insights into *Plasmodium* gametocyte and transmission biology gained using molecular tools over the past decade (Box 1). We suggest approaches to tackle some of the remaining questions and discuss the implications for control measures.

## The ‘growing’ reservoir of infectious gametocytes

Surveys based on the examination of blood smears by microscopy have consistently shown that gametocytes are observed only in a subset of infected patients [7]. However, malaria parasites can transmit from these supposedly ‘non-infectious’ hosts, and the presence of gametocytes at extremely low densities has long been suspected [5]. Molecular-amplification-based techniques are sensitive enough to detect and quantify gametocytes at very low densities (e.g. 0.02–10 gametocytes per microlitre [12]) and have confirmed the presence of gametocytes at densities too low to be detected by standard microscopy methods. We refer to gametocyte densities that are unlikely to be detected by microscopy but can be detected by molecular methods as ‘submicroscopic’. Submicroscopic gametocyte carriers exist at a higher frequency than expected and at all levels of endemicity [12,19–25], and, most importantly, make considerable contributions to transmission [26]. The successful infection of vectors is generally positively correlated to gametocyte density [26–31], but the probability of mosquito infection at low gametocyte densities is higher than that predicted by simple linear relationships. This raises the possibility that gametocytes can cluster in the capillaries [32–34] or that per-gametocyte infectivity is density-dependent. The second scenario could occur if transmission-blocking immune factors are more likely to be found in bloodmeals from high-density infections [33].

In addition to the density and sex ratio of gametocytes, transmission success is also influenced by the timing of gametocyte production. Molecular assays have revealed that this is of particular importance in areas with seasonal transmission because asymptomatic infections with submicroscopic gametocyte densities are likely to be the source of annual epidemics in these areas [20,35]. These infections continue to produce gametocytes at low levels throughout the transmission-free season, and parasites might be capable of increasing the gametocyte conversion rate in response to cues that indicate the start of the transmission season. There are many cues that parasites could use as correlates of the transmission season, but studies testing whether parasites respond to the presence of mosquitoes probing their hosts (for example, detecting factors produced from mosquito salivary glands) have given mixed results. There is some support for the hypothesis that parasites of the rodent malaria *Plasmodium chabaudi* increase investment in gametocytes [36], but no change was observed when this experiment was repeated in a larger-scale study with *P. chabaudi* and *Plasmodium vinckei* [37]. It is not yet clear why parasites in these chronic infections continue to produce gametocytes throughout the transmission-free season [19,20]. Investigating whether the conversion to gametocytes during the transmission-free season is a constraint (by-product) of the cell cycle or an adaptation is a key question. Understanding how host immune responses regulate parasitaemia in chronic infections might help to explain patterns of gametocyte investment. For example, investing in gametocytes could ensure that the asexual parasite density stays low during recrudescence, which might enable parasites to keep their total numbers below the threshold for the reactivation of the immune system. Alternatively, these chronic infections often comprise multiple genotypes, and competition between parasites could shape the patterns of gametocyte investment [38].

## Hostile in-host environmental conditions

An increase in gametocyte conversion has been observed in response to drug therapy and various forms of host anaemia [16,39–46]. Gametocyte conversion is increased when reticulocytes (immature erythrocytes) are added to *P. falciparum* culture [46] and, in the rodent malarias *P. chabaudi* and *Plasmodium berghei,* when reticulocyte release and production is stimulated by administering phenylhydrazine to their hosts [47]. More recently, experimental infections have implicated erythropoietin (EPO), a hormone produced by anaemic hosts, as a possible parasite cue for gametocyte conversion and sex-ratio alteration [43,44]. Because correlations between gametocyte density and reticulocyte density (positive correlation) or haematocrit (negative correlation) have been documented in natural [48] and experimental infections [49], elucidating whether parasites are responding to EPO or reticulocytes is necessary to determine what factors parasites monitor throughout their infections. Intriguingly, *P. chabaudi,* a species that can infect reticulocytes, responds to EPO by upregulating conversion, but *P. vinckei,* which only infects mature erythrocytes, responds by altering its sex ratio instead. This indicates that the host-cell preference of parasite species also influences their patterns of conversion. These problems could be tackled by using molecular assays to detect young gametocytes and accurately quantify conversion.

Elevated gametocyte densities after exposure to anti-malarial drugs have been observed in the rodent malarias *P. chabaudi* and *P. vinckei* [40–42] and in the human malaria *P. falciparum* [12,21,45,50–55]. In the rodent malarias, this response is thought to be due to increased gametocyte conversion, but in *P. falciparum,* post-treatment peaks of gametocyte density often occur sooner than the development time required for a new cohort to appear. Molecular methods have provided data [12,53] to support the hypothesis [45] that the drug-induced release of sequestered gametocytes occurs, rather than an increase in conversion. Whether mature (infectious) or immature (non-infectious) gametocytes are released from sequestration is not known, but this could be investigated by monitoring post-treatment gametocyte cohorts by using molecular assays that detect transcripts of early- and late-stage gametocytes. Furthermore, whether this variation in sequestration time represents a by-product of drug action or is an adaptive shortening of development time remains to be tested. Why should rodent, but not human, malaria parasites increase conversion? Clues could lie in the considerable difference in the gametocyte development time of these taxa. Rodent malaria gametocytes take 36–48 h to reach maturity, so these species might be able to produce a timely response to the rapid action of drugs, whereas the 7–10 days that are required for *P. falciparum* gametocytes to develop might constrain their ability to keep up with rapid or unpredictable drug action and would certainly result in changes to conversion being more difficult to correlate with events in the past.

## Gametocytes in multigenotype infections

Most malaria infections of humans and other animals contain many genotypes [49,56–60] owing to sequential infection in areas of high transmission intensity or the simultaneous acquisition of multiple genotypes from a single mosquito bite. Molecular assays have revealed that individual genotypes within an infection produce gametocytes simultaneously and, in multigenotype infections, this process lasts longer than in single infections [20]. Understanding how the genetic complexity of co-infecting parasites influences gametocyte dynamics and their infectivity to mosquitoes is central to understanding transmission dynamics at the population level. In addition, synthesis of data on the performance of individual genotypes during multi-genotype infections and the probability of their simultaneous transmission to mosquitoes will provide better estimates of recombination rates [61].

In addition to their ability to detect gametocytes at low density, molecular assays also enable the discrimination and quantification of gametocytes produced by different genotypes within the same infection. So far, genotype-specific assays have been developed for *P. falciparum* and *P. chabaudi* gametocytes [15,16]. The application of these assays has revealed that co-infecting genotypes can simultaneously produce gametocytes and transmit, throughout both clinical and asymptomatic *P. falciparum* infections [25]. The presence and density of co-infecting genotypes can fluctuate markedly throughout their infections [35,57] as a result of within-host competition. For example, the genotypes that are best at competing for red blood cells or evading the immune system might have a transmission advantage over their co-infecting competitors. Transmission studies have supported this prediction by showing that although minority genotypes can transmit [62], the transmission of competing genotypes is reduced [63]. Furthermore, when competition occurs between drug-resistant and drug-sensitive genotypes, the resistant genotype tends to be at a disadvantage in the absence of drugs [16].

Laboratory studies using *P. chabaudi* have shown that, in general, virulent (majority) genotypes have a competitive advantage and tend to suppress the growth of less-virulent (minority) competitors [16,63–65]. Faster-growing genotypes could out-compete others through resource competition or by eliciting an immune response that was sufficient to clear minority, but not majority, genotypes, and these two mechanisms are not mutually exclusive. By keeping conversion to gametocytes low, fast-growing genotypes can maximize their investment in asexual replication and, thus, acquire a greater share of host red blood cells than their competitors [38]. Furthermore, genotypes that maintain high asexual densities relative to gametocytes could shelter their gametocytes from strain-specific immune responses acting in the host or minimize the availability of gametocyte antigens that elicit the development of transmission-blocking immune factors. These hypotheses remain to be tested but could reveal that lower gametocyte conversion has evolved in areas with high transmission, when compared to areas of low transmission [38]. However, in *P. chabaudi,* experiments indicate that genotypes do not conditionally alter conversion when in a single- or a double-genotype infection [18,65].

Most laboratory-based studies have focused on gametocyte dynamics during the acute phase of infections, but the transmission advantage of virulent (majority) genotypes might not extend into the chronic phase because the development of anaemia and the immune response could alter the balance between asexual replication and gametocyte density. Quantifying genotype-specific investment into gametocytes during chronic infections in which co-infecting genotypes vary in competitive ability would be useful. Assays designed specifically to detect the gametocytes of minority genotypes will avoid the problem of over-amplifying majority genotypes and, thus, missing the minority ones of interest [66]. However, although genotype-specific assays can be designed for experimental infections with well-identified genotypes, unknown genotypes, which make the identification of suitable genotype-specific assays more difficult, are expected in field studies.

## Sophisticated sex allocation

Successful transmission to vectors is related not only to the density of infectious gametocytes but also to their sex ratio. Sex ratios in malaria parasites are generally female-biased [8], and evolutionary theory predicts that sex ratios reflect the inbreeding rate [67–69]. When taken up in a bloodmeal of a vector, gametocytes must rapidly form gametes, and fertilization can occur between gametes from the same (inbreeding) or different (outbreeding) genotypes. Each male gametocyte can differentiate into up to eight gametes, whereas each female gametocyte differentiates to form a single gamete. Therefore, because males can each fertilize more than one female, a female-biased sex ratio maximizes fertilization success and, thus, represents the most efficient allocation of resources [68,69]. However, this is only the case when the gametocytes in a bloodmeal are genetically related and inbreeding occurs. In this situation, by producing the minimum number of male gametocytes necessary to fertilize the females, parasites are reducing competition for mates between related males and they are maximizing the number of females with which they can mate. Conversely, when infections are composed of several genotypes, the greatest fitness benefits come from increasing investment in male gametocytes. This is because a genotype will gain more fertilizations from each male gametocyte (because each will mate with several females) than from each female (which can be mated only once) [68,69].

The application of this theory to malaria parasites has been rather controversial [8,70] because it does not explain why sex ratios vary throughout infections [18] or why population sex ratios in related Apicomplexans do not correspond to their inbreeding rate (see, for example, Ref. [71]). The strongest support for this theory in other taxa has come from experimental tests that show that individuals can evaluate the inbreeding rate and facultatively adjust their sex ratio accordingly [72]. The recent development of molecular assays that are both genotype- and sex-specific has enabled analogous experiments and revealed that *P. chabaudi* genotypes can evaluate their inbreeding rate and adjust their sex ratio as predicted [49]. More broadly, this also reveals that *P. chabaudi* parasites can discriminate genetically identical clone-mates from con-specific genotypes in their infections. Whether they are able to ‘discriminate kin’ directly (for example, using ‘quorum sensing’) or indirectly (by using some information about their environment, such as the presence of different immune factors) is yet to be investigated.

The use of sex-specific assays to track sex ratios throughout single-genotype infections of *P. chabaudi* has also revealed that patterns of sex allocation during infections correlate with factors such as gametocyte density and host anaemia [49]. Specifically, more males are produced when gametocyte and red blood cell density are low, and reticulocyte density is high. When sex ratios are very female-biased and gametocyte density is low or hosts are anaemic, there is a risk of too few males being taken up in bloodmeals to fertilize the females. Furthermore, even if there are plenty of gametocytes, transmission-blocking immune factors could impair the ability of males to make viable gametes, and the development of such immune factors has been proposed to coincide with anaemia [43]. Therefore, in either of these scenarios, genotypes must ensure that their females are fertilized by investing in extra males (more than expected from their inbreeding rate alone) [8,73]. To date, the experimental work has been focused largely on *P. chabaudi*, so developing assays to test these hypotheses more generally is required. Molecular assays to discriminate different genotypes in natural infections of the lizard malaria, *Plasmodium mexicanum,* have already been developed, and field experiments with this system are possible [74].

## Concluding remarks

Over the past decade, molecular methods have superseded traditional microscopy because they have enabled, for the first time, gametocytes at low densities to be readily quantified and gametocytes produced by parasites of different genotypes in multi-genotype infections to be distinguished. Molecular methods have also facilitated the reliable and rapid detection and quantification of gametocytes at different developmental stages and sexes. Some long-standing questions have been answered and new lines of research have developed.

## Future directions

Gametocytes might no longer be elusive, but many mysteries and challenges remain. For example, despite recent advances in mapping genes that are involved in gametocytogenesis [1], many aspects of gametocyte biology are unclear. One such outstanding question is, ‘How is the sex of gametocytes determined?’ [75]. A better understanding of how sex allocation is influenced by changes in anaemia, immune factors, genotype multiplicity and variable environmental factors (such as mosquito abundance and drug pressure) could provide valuable clues. This could be achieved by integrating expression profiles of a large number of gametocyte- and sex-specific proteins of *Plasmodium* parasites into methods to analyse gametocytes in different stages of development, maturity and sexes. Testing when, why and how gametocyte conversion is shaped by environmental factors and by competition in multi-genotype infections is also necessary. This requires differentiating gametocytes produced by different genotypes, which is a considerable challenge in natural infections. However, a combination of several gametocyte-specific quantification assays based on unlinked single-copy polymorphic genes will reduce the probability of underestimating the number of gametocyte-producing genotypes in natural infections. By continuing to develop and refine molecular methods, we expect that the next decade will reveal a deeper understanding of gametocyte biology and of the mating biology of gametes.

A better understanding of factors that influence gametocyte investment and transmission is essential to the development and evaluation of clinical interventions that disrupt sexual reproduction in *Plasmodium*. For example, asymptomatic infectious individuals are of major importance from a public health perspective. The identification of ‘gametocyte carriers’ and factors that can lead to increased transmission has been considered very important for determining the sources of infection in a community. With many asymptomatic carriers contributing to transmission, the feasibility and public health impact of gametocyte-targeted control, such as intermittent preventive treatment and mass treatment [23,76,77] might need to be revised. Especially in areas with marked seasonal transmission, a reduction of the infectious reservoir over consecutive years could reduce the basic reproduction rate (*R*_0_; the number of future cases derived from one infective case at the present time) to a controllable level. However, a limitation to this approach in the short term is the presence of drug-resistant parasites that can overcome the effect of drugs used and escalate in frequency in the face of drug pressure [76,77]. For the determination of malaria-control strategies, outcomes related to transmission, including post-intervention gametocyte prevalence and density, should be assessed and measures to counter increased gametocytaemia should be considered. Therefore, molecular gametocyte detection and quantification should be added to existing protocols for the evaluation of control strategies, including anti-malarial drugs efficacy [78].

## Box 1 Developments and discoveries made during a decade of molecular gametocyte detection

**1999** Qualitative detection of *P. falciparum* gametocytes by reverse transcriptase (RT)-PCR confirms the presence of submicroscopic gametocytes [9].

**2000** Qualitative genotype-specific RT-PCR to detect *P. falciparum* gametocytes reveals that multiple co-infecting genotypes within an infection can simultaneously produce gametocytes [15].

**2002** RT-PCR used to show that gametocytes at submicroscopic densities are present in infections during the transmission-free season in Sudan [19].

**2004** Quantitative nucleic acid sequence-based amplification (NASBA) developed to distinguish *P. falciparum* gametocytes at early and late stages of development reveals that gametocytes are produced soon after parasites are released from the liver [14].

**2005** Genotype-specific RT-PCR reveals that multi-genotype infections of *P. falciparum* persist and produce gametocytes for longer during transmission-free season than single-genotype infections [20].

**2006** Real-time quantitative assays developed to quantify gametocyte densities for *P. falciparum* [12] and *P. chabaudi* [13]. In *P. falciparum*, gametocytes at submicroscopic densities are common post-treatment and these infections contribute considerably to transmission [12,21]. Previous beliefs, based on microscopic data, are that treatment can result in increased gametocyte prevalence. Molecular assays show that this post-treatment peak in gametocyte prevalence reflects an increase in gametocyte density to levels that are more likely detected by microscopy [12,53]. Furthermore, artemisinin-based combination therapy (ACT) reduces gametocyte density and the proportion of mosquitoes infected but does not affect the proportion of patients with infectious gametocytes (infectious reservoir) [12,21].

**2007** Real-time RT-PCR reveals that *P. chabaudi* genotypes do not alter their gametocyte conversion rate in response to competition with a co-infecting genotype [65]. However, when competition occurs between a drug-resistant and a drug-sensitive genotype and infections are treated to clear the sensitive genotype, the resistant genotype achieves higher gametocyte densities than when in single-genotype infections [16]. Quantitative genotype-specific and sex-specific RT-PCR developed to quantify *P. chabaudi* gametocyte densities and sex ratios of individual genotypes in multi-genotype infections [18]. In *P. falciparum,* more gametocyte carriers are detected using QT-NASBA than microscopy in Burkina Faso [22] and Tanzania [23,24], and submicroscopic gametocytes contribute to transmission [26]. Primaquine treatment can clear all gametocytes remaining after treatment with ACT and sulphadoxine–pyrimethamine [23].

**2008** Quantitative genotype-specific and sex-specific RT-PCR for *P. chabaudi* reveals: genetic variation for patterns of sex-ratio variation observed throughout infections; sex-ratio patterns correlate with host anaemia, and total parasite and gametocyte density; and genotypes increase their investment in male, relative to female, gametocytes in response to the presence of co-infecting genotypes. Genotype-specific RT-PCR for *P. falciparum* reveals that minority genotypes in multiple-clone infections are able to transmit to mosquitoes [25]. In mixed-species infections with *Plasmodium malariae, P. falciparum* gametocyte densities are higher than in single-species infections [79].
